# NaF PET/CT for response assessment of prostate cancer bone metastases treated with single fraction stereotactic ablative body radiotherapy

**DOI:** 10.1186/s13014-019-1359-0

**Published:** 2019-09-05

**Authors:** Nicholas Hardcastle, Michael S. Hofman, Ching-Yu Lee, Jason Callahan, Lisa Selbie, Farshad Foroudi, Mark Shaw, Sarat Chander, Andrew Lim, Brent Chesson, Declan G. Murphy, Tomas Kron, Shankar Siva

**Affiliations:** 1 Department of Physical Sciences, Peter MacCallum Cancer Centre, Melbourne, VIC 3000 Australia; 2 Division of Cancer Imaging, Peter MacCallum Cancer Centre, Melbourne, VIC 3000 Australia; 3 Division of Radiation Oncology, Peter MacCallum Cancer Centre, Melbourne, VIC 3000 Australia; 4 Olivia Newton-John Cancer & Wellness Centre | Austin Health, 145 Studley Road, PO Box 5555, Heidelberg, 3084 Australia; 5 Department of Radiation Therapy, Peter MacCallum Cancer Centre, Melbourne, VIC 3000 Australia; 6 Cancer Surgery, Peter MacCallum Cancer Centre, Melbourne, VIC 3000 Australia; 7 Centre for Medical Radiation Physics, University of Wollongong, Wollongong, NSW 2522 Australia; 8 Sir Peter MacCallum Department of Oncology, University of Melbourne, Parkville, VIC 3000 Australia

**Keywords:** Prostate cancer, Metastases, SABR, Imaging, PET, NaF

## Abstract

**Introduction:**

In prostate cancer patients, imaging of bone metastases is enhanced through the use of sodium fluoride positron emission tomography (^18^F-NaF PET/CT). This imaging technique shows areas of enhanced osteoblastic activity and blood flow. In this work, ^18^F-NaF PET/CT was investigated for response assessment to single fraction stereotactic ablative body radiotherapy (SABR) to bone metastases in prostate cancer patients.

**Methods:**

Patients with bone metastases in a prospective trial treated with single fraction SABR received a ^18^F-NaF PET/CT scan prior to and 6 months post-SABR. The SUV_max_ in the tumour was determined and the difference between before and after SABR determined. The change in uptake in the non-tumour bone was also measured as a function of the received SABR dose.

**Results:**

Reduction in SUV_max_ was observed in 29 of 33 lesions 6 months after SABR (mean absolute decrease in SUV_max_ 17.7, 95% CI 25.8 to − 9.4, *p* = 0.0001). Of the three lesions with increased SUV_max_ post-SABR, two were from the same patient and located in the vertebral column. Both were determined to be local progression in addition to one fracture. The third lesion (in a rib) was shown to be controlled locally but suffered from a fracture at 24 months. Progression adjacent to the treated volume was observed in two patients. The non-tumour bone irradiated showed increased loss in uptake with increasing dose, with a median loss in uptake of 23.3% for bone receiving 24 Gy.

**Conclusion:**

^18^F-NaF PET/CT for response assessment of bone metastases to single fraction SABR indicates high rates of reduction of osteoblastic activity in the tumour and non-tumour bone receiving high doses. The occurrence of marginal recurrence indicates use of larger clinical target volumes may be warranted in treatment of bone metastases.

**Trial registration:**

POPSTAR, ‘Pilot Study of patients with Oligometastases from Prostate cancer treated with STereotactic Ablative Radiotherapy’, Universal Trial Number U1111-1140-7563, Registered 17th April 2013.

**Electronic supplementary material:**

The online version of this article (10.1186/s13014-019-1359-0) contains supplementary material, which is available to authorized users.

## Background

Prostate cancer represents a major cancer burden in men, representing the most common male cancer diagnosis [1]. Prostate cancer staging determines appropriate treatment at initial presentation and during disease progression and makes use of various medical imaging techniques. The most probable site of distant metastases in prostate cancer is bone, thus imaging techniques used for visualization of prostate metastases must be able to accurately visualize sites of bone disease [2]. This is particularly so with increasing interest in metastasis-directed therapy (MDT) for oligometastatic prostate cancer [3–5]. The standard imaging for determination of prostate cancer bone metastases has been whole body bone scan with 2D scintigraphy or single photon emission computed tomography (SPECT) approaches, using ^99m^Tc methylene diphosphonate [6, 7]. These tracers are taken up at sites of high osteoblastic activity representing bone turnover. The tumour burden as represented on bone scans can be quantified into a bone scan index (BSI), which has been shown as an independent prognostic marker for survival [8, 9]. Bone scans have many limitations however, such as poor anatomical correlation and low specificity and sensitivity [10, 11].

Prior to use of ^99m^Tc MDP, ^18^F sodium fluoride (^18^F-NaF) was used for planar scintigraphy [12]. In recent years however ^18^F-NaF has been used for PET/CT acquisition, which allows high spatial resolution 3D imaging of osteoblastic activity and blood flow [13–15]. ^18^F-NaF has been shown to have improved sensitivity and specificity for prostate cancer metastases, compared with ^99m^Tc MDP [10], although improvements through use of quantitative SPECT have recently suggested consistent standardised uptake value (SUV) between the two modalities for prostate and breast bone metastases [16].

In the context of oligometastatic prostate cancer, ^18^F-NaF PET/CT imaging facilitates high quality detection and visualization of skeletal metastases which may be suitable for local MDT such as stereotactic ablative body radiotherapy (SABR). In this study we examine ^18^F-NaF uptake prior to and after single fraction SABR to bone metastases in patients enrolled in a prospective clinical trial. We investigate ^18^F-NaF uptake in tumour and non-tumour bone, with the hypothesis that tumour and normal tissue response to SABR can be assessed by ^18^F-NaF PET/CT.

## Methods

This is a pre-specified exploratory analysis of a prospective clinical trial (POPSTAR, ‘Pilot Study of patients with Oligometastases from Prostate cancer treated with STereotactic Ablative Radiotherapy’, Universal Trial Number U1111–1140-7563) [17]. Between April 2013 and November 2014 33 patients with oligometastic prostate cancer were enrolled with written informed consent. They received a single fraction of 20 Gy to a total of 50 metastases. All lesions in a given patient were treated synchronously within in a single treatment course. All patients had a ^18^F-NaF PET/CT at screening, and 6 months post-treatment. Patients were excluded from the study if they had more than three metastases after PET/CT screening. The Quality of Life including pain scores, and disease progression for the whole cohort has previously been reported [17]. The current analysis is limited to those patients with demonstrable bone metastases who received the treatment protocol.

3 MBq/kg of ^18^F-NaF was administered by intravenous injection followed by a 60 min uptake period. A low-dose CT acquisition was obtained first followed by the PET acquisition. Patients were imaged from vertex to toes on a PET/CT scanner (Discovery 690 GE Healthcare, USA). No fasting was required. Patients were encouraged to void prior to imaging.

Radiotherapy simulation CT was performed less than 2 weeks prior to radiotherapy treatment. Scans were performed on a Philips Brilliance Big Bore CT scanner with a 2 mm slice thickness at 140 kV. The gross tumour volume (GTV) was contoured as visualised on the ^18^F-NaF PET and planning CT imaging, limited to bone. For non-verterbral metastases, an isotropic 5 mm planning target volume (PTV) margin was applied to the GTV to account for geometric uncertainties in the treatment. In the case of vertebral metastases, a clinical target volume (CTV) was applied according to the International Spine Radiosurgery Consortium consensus guidelines [18]. A 2–3 mm PTV margin was then applied to the CTV. Treatment planning was performed in the Eclipse treatment planning system (v11, Varian Medical Systems, Palo Alto, USA). Non-vertebral targets were treated with 3D conformal treatment plans which consisted of 7–9 beams typically including 1–2 non-coplanar beams. Dose was prescribed such that at least 99% of the PTV received 20 Gy, with a maximum dose between 125 and 140%. Vertebral targets were treated with a 9–12 coplanar IMRT fields, and prescribed such that at least 80% of the PTV received 18 Gy, with a maximum dose between 125 and 140%. Dose was calculated with the AAA algorithm at 2.5 mm resolution for non-vertebral targets and 1.5 mm resolution for vertebral targets. Radiotherapy was delivered on a Varian 21iX or Varian TrueBeam STx linear accelerator. Patients were immobilised in a vacuum immobilisation bag. Image guidance was performed using cone-beam CT (CBCT) and/or Exactrac planar x-ray imaging with a 0 mm tolerance for shifts. Mid-treatment CBCT was performed to ensure patient setup accuracy during treatment.

### Image response assessment

The pre-treatmentand post-treatment ^18^F-NaF PET/CT scans and the radiotherapy planning CT scan with contours and dose grid were imported into MIM software (v6.6, MIM software, Cleveland, USA). The CT components of the ^18^F-NaF PET/CT scans were registered to the planning CT scan. An initial rigid registration was performed on the whole CT data set. This was further refined by rigid registration using a bounding box approximately 5 × 5 × 5 cm surrounding the GTV. This was manually adjusted if required to ensure accurate registration at the bone target. This rigid registration was then applied to the PET component of the ^18^F-NaF PET/CT scan.

#### Tumour response

The SUV_max_ was determined for the GTV contour from the pre- and post-treatment ^18^F-NaF PET data. The difference in SUV_max_ from pre- to post-treatment was calculated as a percentage of the pre-treatment SUV_max_.

#### Normal bone response

The bone was contoured on the axial slices from 2 cm above to 2 cm below the PTV using a threshold of 120 HU followed by manual correction. An isotropic 2 cm margin was applied to the PTV and the intersection of this and the bone contour was derived to result in a proximal bone (bone within 2 cm of the target). This ensured the bone contour included only the bone that was accurately registered between the three scans. The GTV was subtracted from the proximal bone contour to obtain proximal non-tumour bone. The radiotherapy isodose lines at 2 Gy intervals were converted into contours. These were subtracted from each other, and Boolean intersection with the proximal non-tumour bone was performed to result in contours covering proximal non-tumour bone receiving each 2 Gy dose interval up to 24 Gy. The mean, median, maximum and standard deviation in non-tumour bone receiving each dose interval was extracted. The change in mean SUV after SABR was computed for proximal bone receiving each of the dose levels as [SUV_post_ – SUV_pre_] / SUV_pre_. The change in SUVmean in the non-tumour bone was reviewed for all patients with bone fractures post treatment (CTCAE v4.0).

## Results

Twenty-one patients from the patient population with bone metastases were included in this analysis. A total of 33 bone lesions were irradiated (Additional file 1: Table S1). The baseline SUV characteristics of the lesions is shown in Additional file 2: Table S2. The mean (± 1 st. dev.) time of post-therapy PET was 7.4 ± 1.0 months. The SUV_mean_ of normal, un-irradiated bone was consistent between pre and post-treatment scans, with a mean ratio, post/pre of 0.98 ± 0.08. In this cohort, ^18^F-NaF had detected an additional 14 metastases, over that detected with CT and bone scan.

### SUV_max_ differences in tumour

**Fig. 1:**
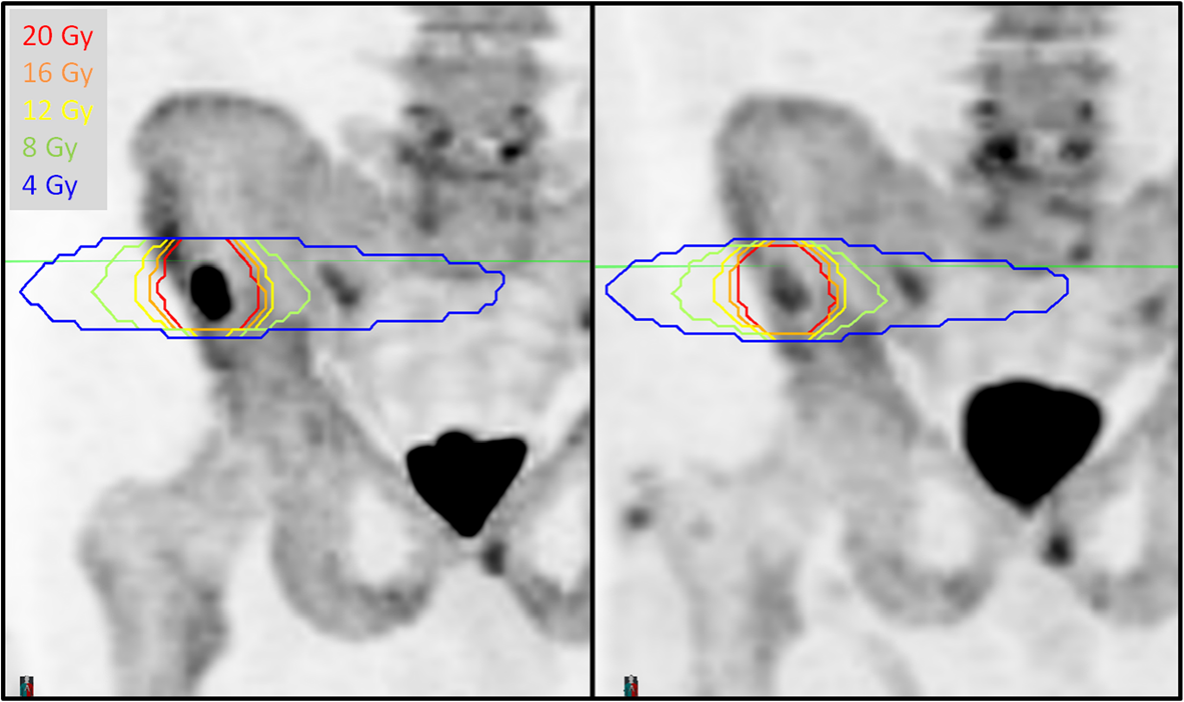
MIP of pre-treatment NaF PET (left) and post-treatment NaF PET for Patient 31. The planned isodose lines from are shown

**Fig. 2:**
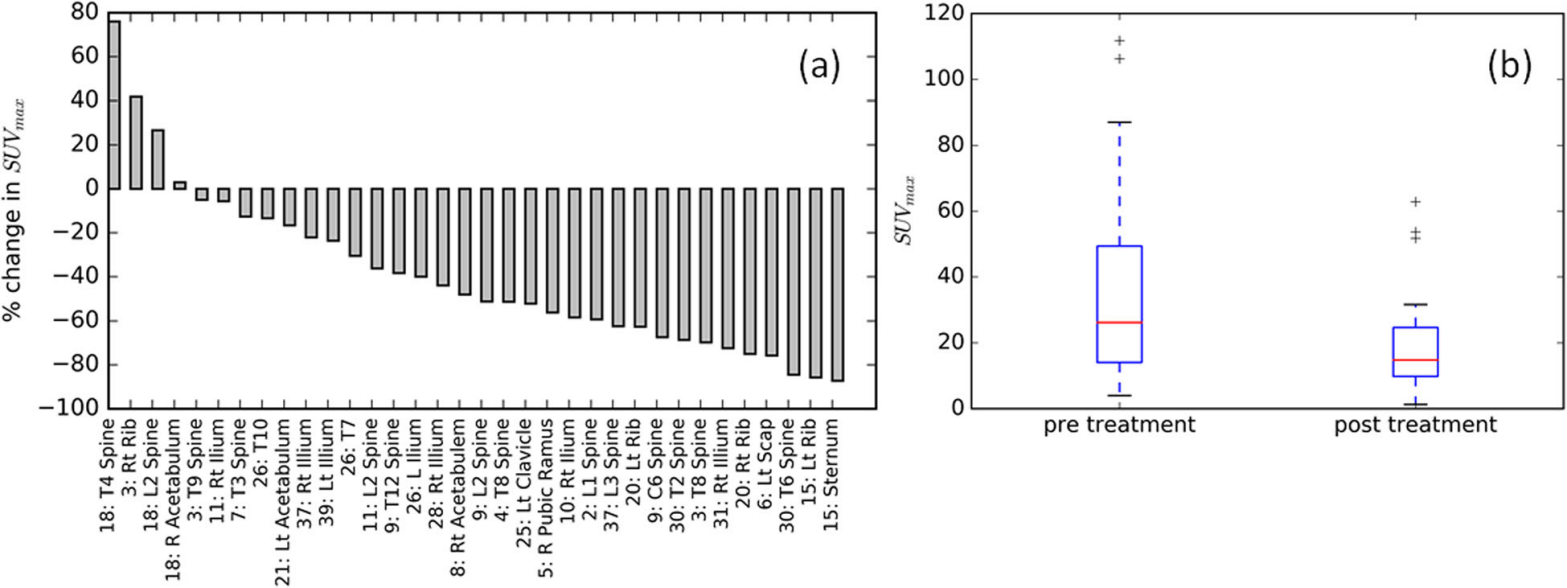
**a** Waterfall plot of the change in SUVmax of the GTV after SABR and (**b**) absolute SUV_max_ before and after SABR

**Fig. 3:**
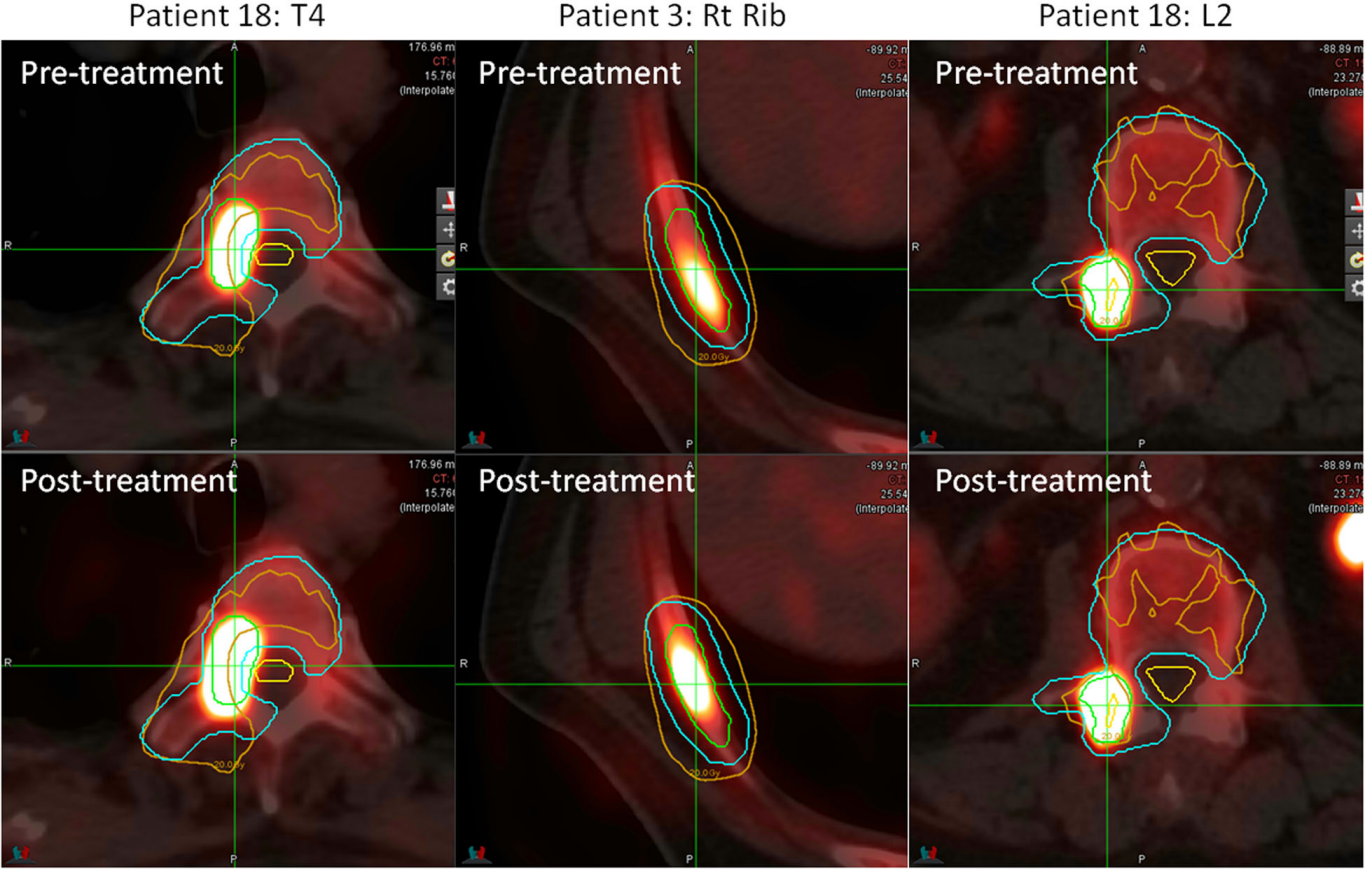
Pre and post-treatment NaF scans for the two patients (three lesions) with increased SUV_max_ in the tumour. The tumour is shown by the green contour, the PTV in blue and the volume receiving 20 Gy in orange. The spinal cord is shown in yellow for Patient 18

The SUV_max_ was computed in the GTV contour on pre and post treatment ^18^F-NaF PET scans. Figure 1 shows for an example patient the maximum intensity projection of the pre and post-SABR ^18^F-NaF PET images with the planned isodose lines. Figure 2 shows a waterfall plot of the relative change in SUV_max_ after SABR. Reduction in tumour SUV_max_ was observed in 29/33 lesions. The mean absolute decrease in SUVmax after SABR was 17.7 (95% CI − 25.8 to − 9.4, *p* = 0.0001). Increase in SUV_max_ at 6 months was observed in three of 33 lesions treated with SABR; of these, two were from the same patient. Figure 3 shows the three lesions with increased SUV_max_. Patient 18 had T4 and L2 lesions that were determined to be a local progression based on follow up with CT and PSMA PET imaging at 20 months post treatment. Specifically for the T4 lesion, full coverage of the GTV with the prescription dose was not achieved due to the proximity of the spinal cord, which was a dose-limiting structure. In addition, a grade 3 fracture was also observed at 18 months post treatment at L2. In the case of a right rib lesion in patient 3, local control based on repeat CT and PSMA PET was achieved; however a grade 2 fracture was observed at 24 months post SABR.

### Non-tumour bone

**Fig. 4:**
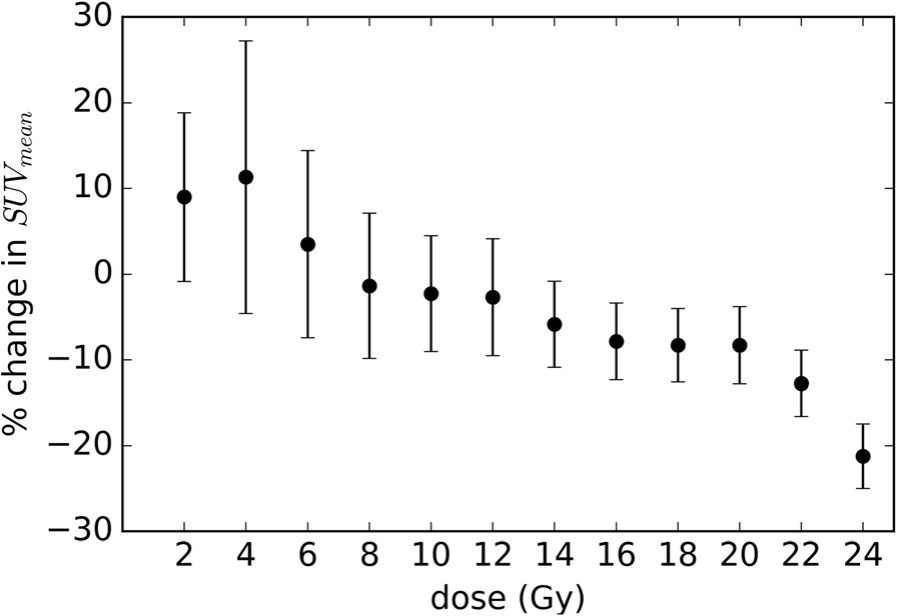
Mean change in SUV_mean_ in non-GTV bone for the population of patients. The uncertainty bars represent ±1 standard deviation

**Fig. 5:**
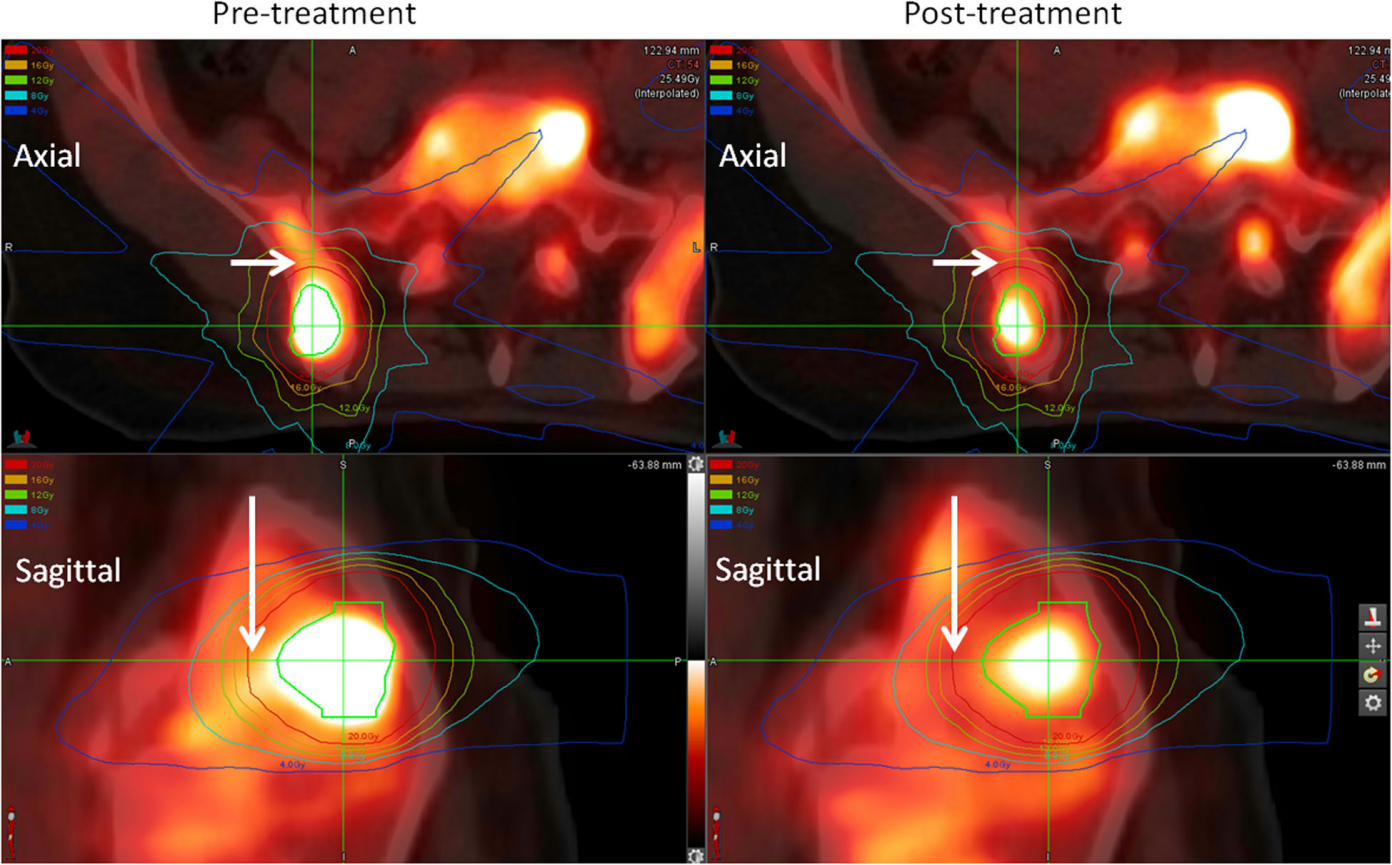
Pre and post-treatment images for Patient 10, Rt Illium. GTV is shown in red, and isodose lines in 2 Gy increments from 4 Gy to 20 Gy are shown various colours. Reduction in the tumour uptake post-treatment is observed, as well as reduction in the non-tumour bone irradiated in particular in the 16–20 Gy region

The change in SUV_mean_ in the non-GTV bone receiving each dose level from 0 Gy to 24 Gy was computed. Figure 4 shows the average change in SUV_mean_ for the non-tumour bone surrounding 33 targets as a function of dose. The mean percentage reduction in the non-tumour bone reduced with increasing dose, with a mean reduction of 23.3% for non-tumour bone receiving 24 Gy. Figure 5 shows a representative patient (Patient 10) treated for a right ilium metastasis. Reduction in the tumour uptake is observed, as is reduction in the surrounding bone uptake in particular at the 16–20 Gy dose range.

**Fig. 6:**
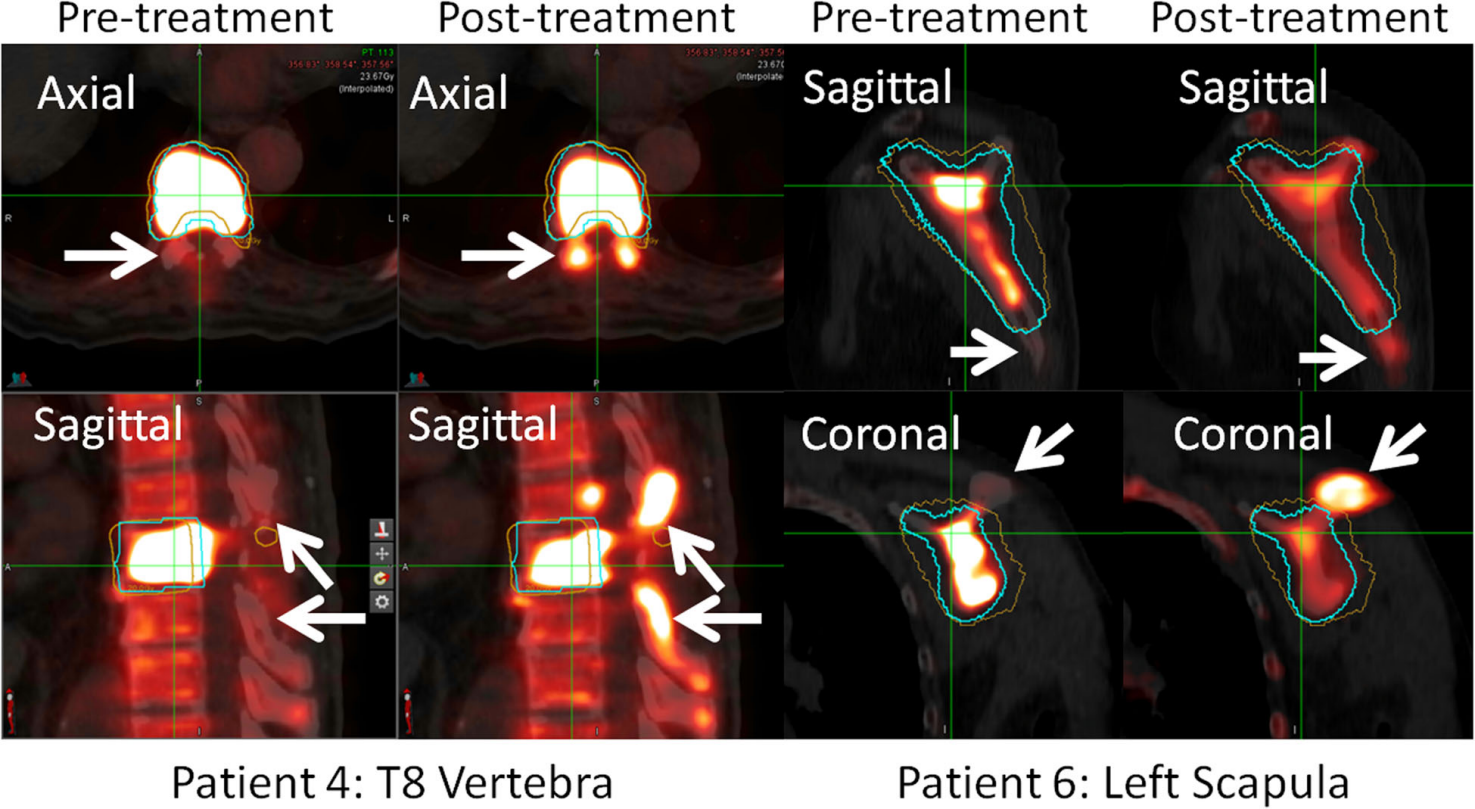
Comparison of (left) baseline and (right) follow-up PET scan of two patients with marginal recurrence. The PTV is shown in blue, and region treated to the prescription dose of 20 Gy is shown in orange. Increased uptake immediately adjacent to the irradiated region is highlighted

In three patients, markedly higher ^18^F-NaF uptake in the low dose area of non-tumour bone was observed. In Patient 6, although there was significant reduction in uptake in the treated area, increased uptake in the contiguous bone immediately adjacent to the treated volume was observed. Similarly for Patient 4, increased uptake was observed post-treatment immediately adjacent to the treated volume. These were both determined to be marginal recurrence. The pre and post-treatment scans for these two patients are shown in Fig. 6. The third patient with increase in SUV adjacent to the treated volume (Patient 18, R Acetabulum), had subsequent CT imaging which showed stable morphology and PSMA PET negativity.

Two patients had grade 2 fractures (Patient 3: Right Rib and Patient 20: Left Rib) and one patient had a grade 3 fracture (Patient 18, L2 Vertebra). The lesions for Patient 3 and Patient 18 did not show response to treatment at 6 months on the ^18^F-NaF.

## Discussion

The POPSTAR trial was a prospective evaluation of SABR for oligometastases from prostate cancer and involved high, single fraction doses to bone and lymph node metastases [17]. This is the first study to demonstrate ^18^F-NaF as a response assessment tool in the context of SABR to bone metastases. ^18^F-NaF provides high spatial resolution and high sensitivity/specificity measurement of bone metastases and is representative of osteoblastic activity and blood flow. In the current study we have demonstrated that a single high dose fraction of external beam radiotherapy reduces ^18^F-NaF uptake in bone at 6 months, thus can be considered to reduce osteoblastic activity in bone metastases in the majority of patients. We have also shown a reduction of osteoblastic activity in regions of non-tumour bone treated to high doses per fraction. Bone fracture remains a side effect in SABR to bone lesions, with two Grade 2 and one Grade 3 fractures observed in the POPSTAR trial. In all the patients that had bone fracture post-SABR, there was high residual NaF uptake within the treated field, or there was new uptake adjacent to the treated volume as visualised on the follow up ^18^F-NaF PET scan. As reported previously for this cohort, for the bone metastasis-specific Quality of Life module (EORTC QLQ-BM22), painful sites, pain characteristics, and functional interference increased from baseline only at the 24-mo timepoint [17].

In the POPSTAR trial, bone metastasis was defined using a combination of CT and ^18^F-NaF scan information. The primary tumour was delineated by an experienced radiation oncologist and a 5 mm margin was applied to account for geometric uncertainty in the treatment delivery (planning target volume, [PTV]). In the subset of vertebral tumours, a CTV was applied according to international consensus guidelines, with a 2 mm CTV-PTV expansion. The use of ^18^F-NaF PET in the current study however has shown the potential inadequacy of direct expansion to PTV in non-vertebral bone without a CTV margin. Three patients had regions of increased uptake immediately adjacent to the treated volume, suggesting marginal failure. This may be mitigated by the use of a CTV margin for all bone metastases, similar to the international consensus guidelines for spine metastases. Despite the inclusion of a CTV in vertebral targets, there are still limitations with full coverage of the GTV due to the close proximity of dose limiting structures such as the spinal cord.

In more recent years, 18F-Fluoromethylcholine has been used for directing SABR to prostate cancer metastase, with some prognostic value [19]. Prostate specific membrane antigen (PSMA) PET scanning has however become the scanning modality of choice for prostate cancer in the metastatic setting [20]. PSMA PET has been shown to have comparable sensitivity and specificity as ^18^F-NaF PET for bone metastases in one study [21], however Uprimny et al. [22] found ^18^F-NaF PET detected more skeletal metastases and had a higher tumour to background ratio than PSMA PET. PSMA PET has the additional advantage of visualising soft-tissue metastases, however is limited to prostate cancer and renal cell carcinoma [23]. ^18^F-NaF PET thus has a role in response assessment for skeletal metastases in both prostate and non-prostate cancer.

## Conclusion

In this study we have shown the response to a high dose single fraction SABR treatment to prostate bone oligometastases as visualised on ^18^F-NaF PET/CT. In the majority of patients, SABR reduces ^18^F-NaF PET/CT uptake in the tumour, and in high dose regions reduces uptake in non-tumour bone. Regions of increased uptake immediately adjacent to treated volumes suggest that increased clinical target volumes are required in the treatment of bone metastases with SABR.

## Additional files

Additional file 1: **Table S1.** Baseline characteristics of the patients included in this study. These are only the patients that had bone metastases. (DOCX 27 kb)

Additional file 2: **Table S2.** Individual lesion characteristics. Non-contiguous patient numbering is used as we are describing the bone metastases only, rather than all metastases treated in this clinical trial. (DOCX 17 kb)

## Data Availability

The datasets generated and/or analysed during the current study are not publicly available due to consent not obtained to make data public, but are available from the corresponding author on reasonable request, and subject to ethics approval.
