# Supplementary material for: NaF PET/CT for response assessment of prostate cancer bone metastases treated with single fraction stereotactic ablative body radiotherapy
Source: Radiat Oncol. 2019 Sep 5;14:164. doi: 10.1186/s13014-019-1359-0 (PMC6728984; doi:10.1186/s13014-019-1359-0)
Supplement: Supplementary file 1 — Table S1. Baseline characteristics of the patients included in this study. These are only the patients that had bone metastases. (DOCX 27 kb) [file 13014_2019_1359_MOESM1_ESM.docx]

Table S1: Baseline characteristics of the patients included in this study. These are only the patients that had bone metastases.

| **Variable** | **statistic** | **n (%)** |
| --- | --- | --- |
| Age | Mean (SD) | 72 (8) |
|  | Median [range] | 72 [52 - 85] |
| Gleason score | 4 | 1 (5%) |
|  | 6 | 1 (5%) |
|  | 7 | 9 (45%) |
|  | 8 | 8 (40%) |
|  | 9 | 1 (5%) |
| Time from primary cancer treatment to SABR (years) | Mean (SD) | 5.2 (4.0) |
|  | Median [range] | 4.1 [0.7 - 13.9] |
| ECOG | 0 | 16 (80%) |
|  | 1 | 4 (20%) |
| Spine Instability Score (metastasis 1) | 1 | 3 (30%) |
|  | 2 | 1 (10%) |
|  | 3 | 2 (20%) |
|  | 6 | 4 (40%) |
|  | No spine metastasis | 10 |
| Spine Instability Score (metastasis 2) | 1 | 1 (20%) |
|  | 2 | 1 (20%) |
|  | 3 | 1 (20%) |
|  | 5 | 1 (20%) |
|  | 6 | 1 (20%) |
|  | No 2^nd^ spine metastasis | 15 |
| Spine Instability Score (metastasis 3) | 4 | 1 (100%) |
|  | No 3^rd^ spine metastasis | 19 |
| Long bone metastasis | No | 20 (100%) |
|  | Yes | 0 (0%) |
| Bone lesions | 1 | 11 (55%) |
|  | 2 | 4 (20%) |
|  | 3 | 5 (25%) |
| Total lesions | 1 | 11 (55%) |
|  | 2 | 4 (20%) |
|  | 3 | 5 (25%) |
