# Supplementary material for: NaF PET/CT for response assessment of prostate cancer bone metastases treated with single fraction stereotactic ablative body radiotherapy
Source: Radiat Oncol. 2019 Sep 5;14:164. doi: 10.1186/s13014-019-1359-0 (PMC6728984; doi:10.1186/s13014-019-1359-0)
Supplement: Supplementary file 2 — Table S2. Individual lesion characteristics. Non-contiguous patient numbering is used as we are describing the bone metastases only, rather than all metastases treated in this clinical trial. (DOCX 17 kb) [file 13014_2019_1359_MOESM2_ESM.docx]

Table S2: Individual lesion characteristics. Non-contiguous patient numbering is used as we are describing the bone metastases only, rather than all metastases treated in this clinical trial.

| Patient | Lesion | Baseline SUV_max_ | Baseline SUV_mean_ |
| --- | --- | --- | --- |
| POP-02 | L1 Spine | 77.8 | 22.3 |
| POP-03 | T8 Spine | 21.9 | 7.8 |
|  | T9 Spine | 15.3 | 11.8 |
|  | Rt Rib | 11.8 | 5.4 |
| POP-04 | T8 Spine | 106.3 | 41.1 |
| POP-05 | R Pubic Ramus | 49.8 | 14.9 |
| POP-06 | Lt Scap | 111.8 | 29.5 |
| POP-07 | T3 Spine | 26.7 | 14.6 |
| POP-08 | Rt Acetabulem | 51.6 | 14.9 |
| POP-09 | T12 Spine | 25.8 | 12.8 |
|  | L2 Spine | 24.2 | 16.6 |
|  | C6 Spine | 69.2 | 31.2 |
| POP-10 | Rt Ilium | 25.8 | 13.1 |
| POP-11 | L2 Spine | 48.5 | 12.4 |
|  | Rt Ilium | 13.3 | 8.8 |
| POP-15 | Sternum | 87.0 | 26.3 |
|  | Lt Rib | 34.6 | 7.0 |
| POP-18 | T4 Spine | 30.5 | 14.5 |
|  | L2 Spine | 49.7 | 20.3 |
|  | R Acetabulum | 9.8 | 5.6 |
| POP-20 | Lt Rib | 40.4 | 18.2 |
|  | Rt Rib | 38.8 | 18.3 |
| POP-21 | Lt Acetabulum | 13.6 | 6.2 |
| POP-25 | Lt Clavicle | 15.7 | 5.9 |
| POP-26 | T7 Spine | 10.2 | 8.9 |
|  | T10 Spine | 18.2 | 12.0 |
|  | L Ilium | 11.2 | 8.2 |
| POP-28 | Rt Illium | 9.9 | 5.6 |
| POP-30 | T2 Spine | 4.0 | 3.7 |
|  | T6 Spine | 9.2 | 6.6 |
| POP-31 | Rt Illium | 38.4 | 15.4 |
| POP-37 | Rt Illium | 21.1 | 12.8 |
|  | L3 Spine | 74.4 | 40.5 |
| POP-39 | Lt Illium | 33.0 | 17.8 |
